# Transport of ultrasmall gold nanoparticles (2 nm) across the blood–brain barrier in a six-cell brain spheroid model

**DOI:** 10.1038/s41598-020-75125-2

**Published:** 2020-10-22

**Authors:** Viktoriya Sokolova, Gehad Mekky, Selina Beatrice van der Meer, Michael C. Seeds, Anthony J. Atala, Matthias Epple

**Affiliations:** 1 Inorganic Chemistry and Center for Nanointegration Duisburg-Essen (CeNIDE), University of Duisburg-Essen, 45117 Essen, Germany; 2 Wake Forest Institute for Regenerative Medicine, Wake Forest School of Medicine, Winston-Salem, NC 27101 USA; 3 Zoology Department, Faculty of Science, Zagazig University, Zagazig, Egypt

**Keywords:** Blood-brain barrier, Nanobiotechnology, Nanomedicine, Nanoscale materials, Nanotoxicology

## Abstract

The blood–brain barrier (BBB) is an efficient barrier for molecules and drugs. Multicellular 3D spheroids display reproducible BBB features and functions. The spheroids used here were composed of six brain cell types: Astrocytes, pericytes, endothelial cells, microglia cells, oligodendrocytes, and neurons. They form an in vitro BBB that regulates the transport of compounds into the spheroid. The penetration of fluorescent ultrasmall gold nanoparticles (core diameter 2 nm; hydrodynamic diameter 3–4 nm) across the BBB was studied as a function of time by confocal laser scanning microscopy, with the dissolved fluorescent dye (FAM-alkyne) as a control. The nanoparticles readily entered the interior of the spheroid, whereas the dissolved dye alone did not penetrate the BBB. We present a model that is based on a time-dependent opening of the BBB for nanoparticles, followed by a rapid diffusion into the center of the spheroid. After the spheroids underwent hypoxia (0.1% O_2_; 24 h), the BBB was more permeable, permitting the uptake of more nanoparticles and also of dissolved dye molecules. Together with our previous observations that such nanoparticles can easily enter cells and even the cell nucleus, these data provide evidence that ultrasmall nanoparticle can cross the blood brain barrier.

## Introduction

Many diseases affect the human brain. The presence of an intact blood–brain barrier (BBB) is essential to protect the brain against foreign compounds. This is of high clinical relevance for brain tumor imaging and treatment, for stroke treatment, to deliver drugs into the brain, and on a more general level in the area of (nano-)toxicology. Different in vitro models have been developed to investigate the permeability of the BBB, e.g. for drugs. Among these, transwell models and 3D spheroids are most prominent^1–12^. An important question is to what extent nanoparticles are able to cross the blood–brain barrier^11,13–15^, be it desired for therapeutic nanomedical application or after unwanted, unintended exposure to nanoparticles that have entered the bloodstream (nanotoxicology)^16–24^. Only few results have been reported on the transport of inorganic nanoparticles into brain organoids^11,25–28^.

Based on earlier observations that ultrasmall nanoparticles (metallic core diameter about 2 nm) are able to cross cell membranes and even enter the cell nucleus^24,28–31^, we have studied their effect on a well-established and innovative model for the blood–brain barrier, i.e. brain spheroids. These consist of six different cell types that are representative of human brain tissue^8^. It has been shown that they form an intact blood–brain barrier, so that this model can be used as a reproducible in vitro platform to test the permeability of nanoparticles. Here, we have investigated the transport of ultrasmall gold nanoparticles in comparison to a dissolved dye under normoxic and hypoxic conditions as a model of ischemic stroke.

## Results

### Surface-functionalized ultrasmall gold nanoparticles

**Figure 1:**
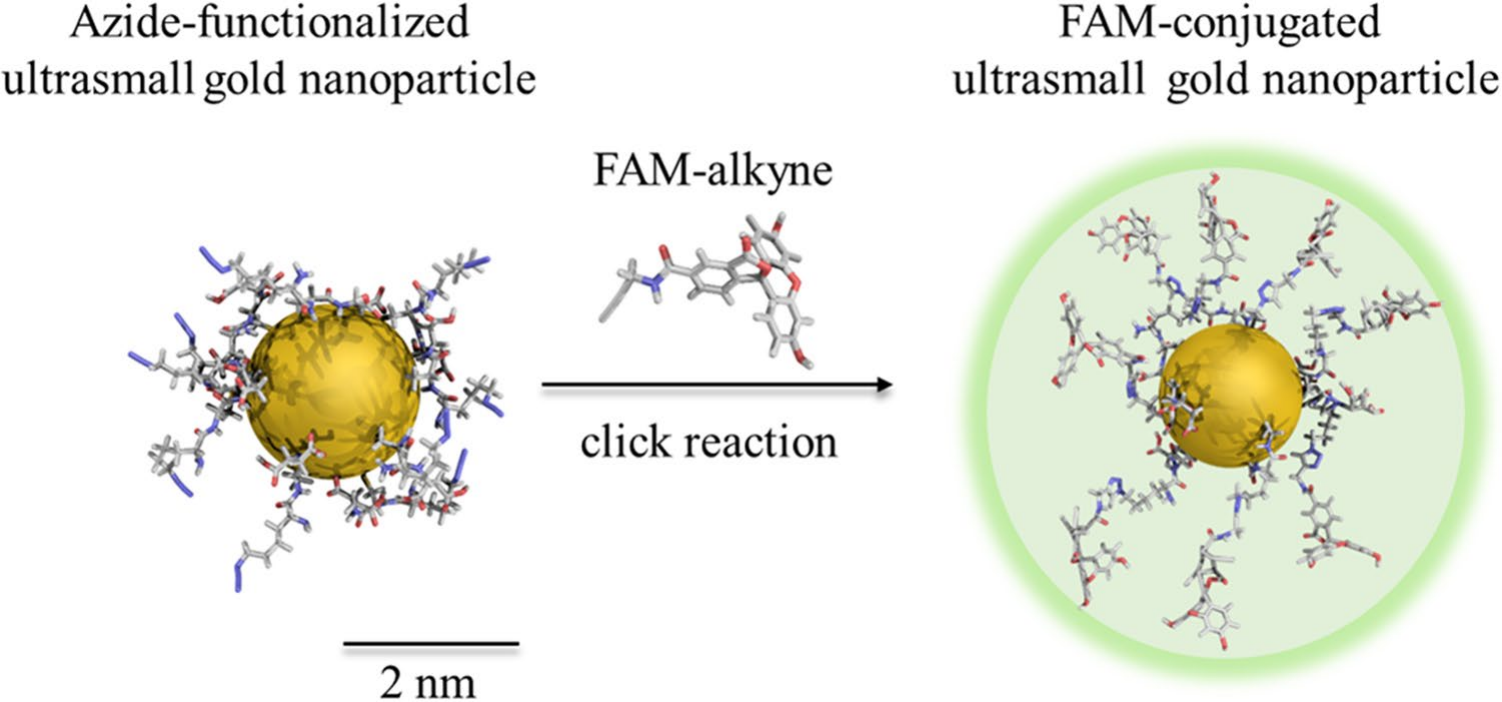
FAM-conjugated ultrasmall gold nanoparticles (schematic representation).

Ultrasmall gold nanoparticles were surface-functionalized with fluoresceine by click reaction as described earlier^30^. The diameter of the spherical gold core was 2.28 ± 0.32 nm when measured by high-resolution transmission electron microscopy (HRTEM). The strong covalent gold-sulfur bond efficiently prevents a dissociation of the dye ligand from the nanoparticle core^28,30,32^. The hydrodynamic diameter is estimated to 3–4 nm, based on ^1^H-DOSY results of similarly functionalized nanoparticles^30^. Figure 1 schematically shows the chemical nature of the nanoparticles.

### Cellular organization of spheroids with barrier formation

**Figure 2:**
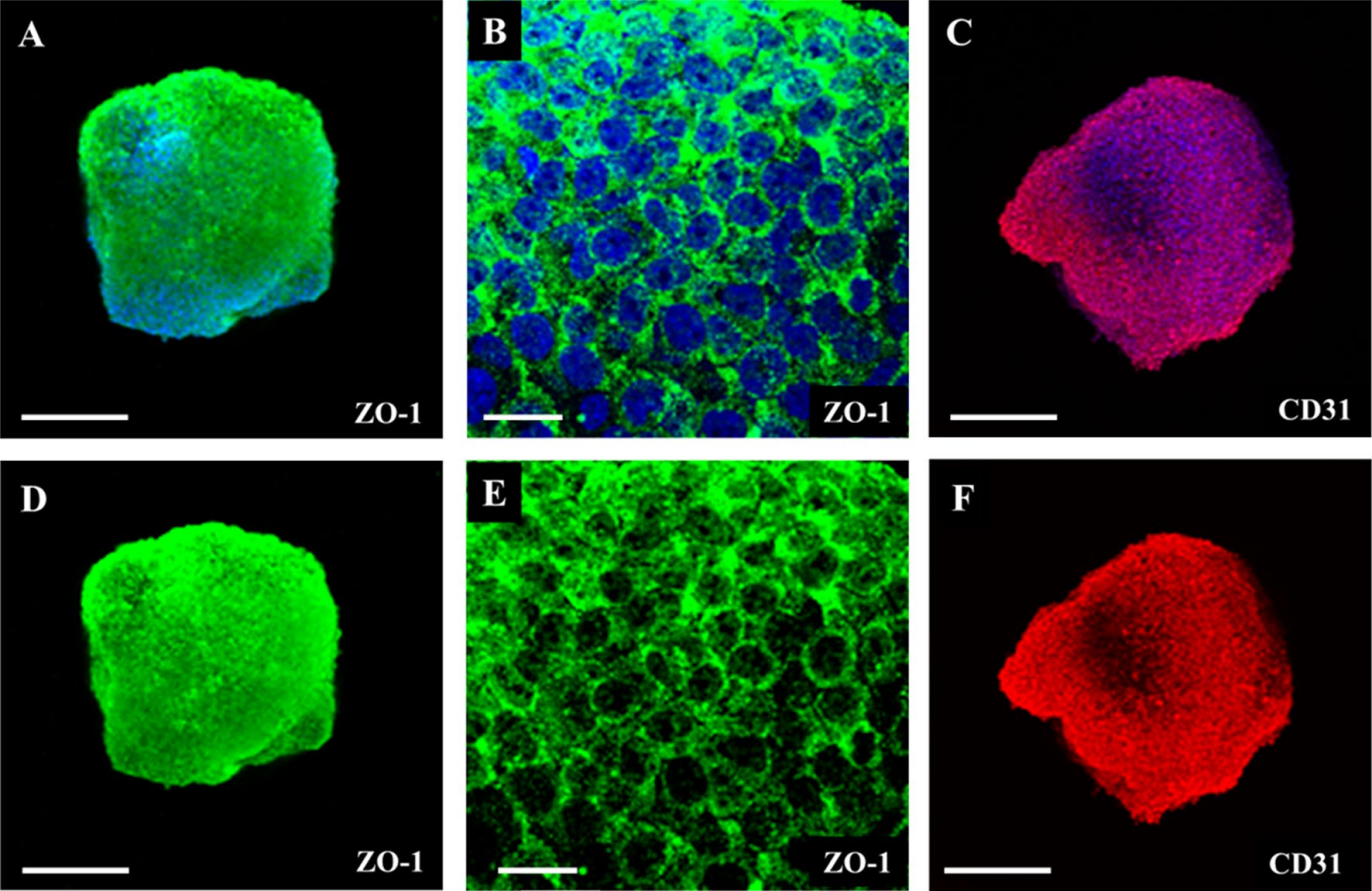
Six cell-type brain spheroid characterization. Immunohistochemistry was conducted on brain spheroids to determine phenotypic protein expression. The spheroids were stained for either ZO-1 or CD31 alone (bottom row) or combined with DAPI nuclear stain (upper row). The selected area was imaged at higher magnification to visualize the cell border connections formed by ZO-1 (**B**,**E**). The CD31 expression shows the localization of HBMEC in the brain spheroids. Images were obtained at × 10 and × 60 (**B**,**E**) magnification. Scale bars 30 μm and 200 μm (for × 10 and × 60, respectively).

The 3D brain spheroids were prepared and characterized according to Nzou et al.^8^ They consisted of six different types of brain cells. First, astrocytes, microglia cells, oligodendrocytes, and neurons were cultivated for 48 h. Then, an outer layer of pericytes and endothelial cells was added. The spheroids were cultivated for another 5 to 6 days before incubation with nanoparticles. Spheroids made by this staged assembly have shown a homogeneous distribution of astrocytes, neurons, microglia and oligodendrocytes and pericytes with a surface layer of CD31 expressing endothelial cells^8,33^. The BBB was formed in the outer layer, as shown by immunohistochemistry (ZO-1 and CD31) and suitable permeability assays^33^. The diameter of a spheroid was about 500 µm at this time point. Figure 2 shows the characterization of the BBB in the six cell-type brain spheroids.

**Figure 3:**
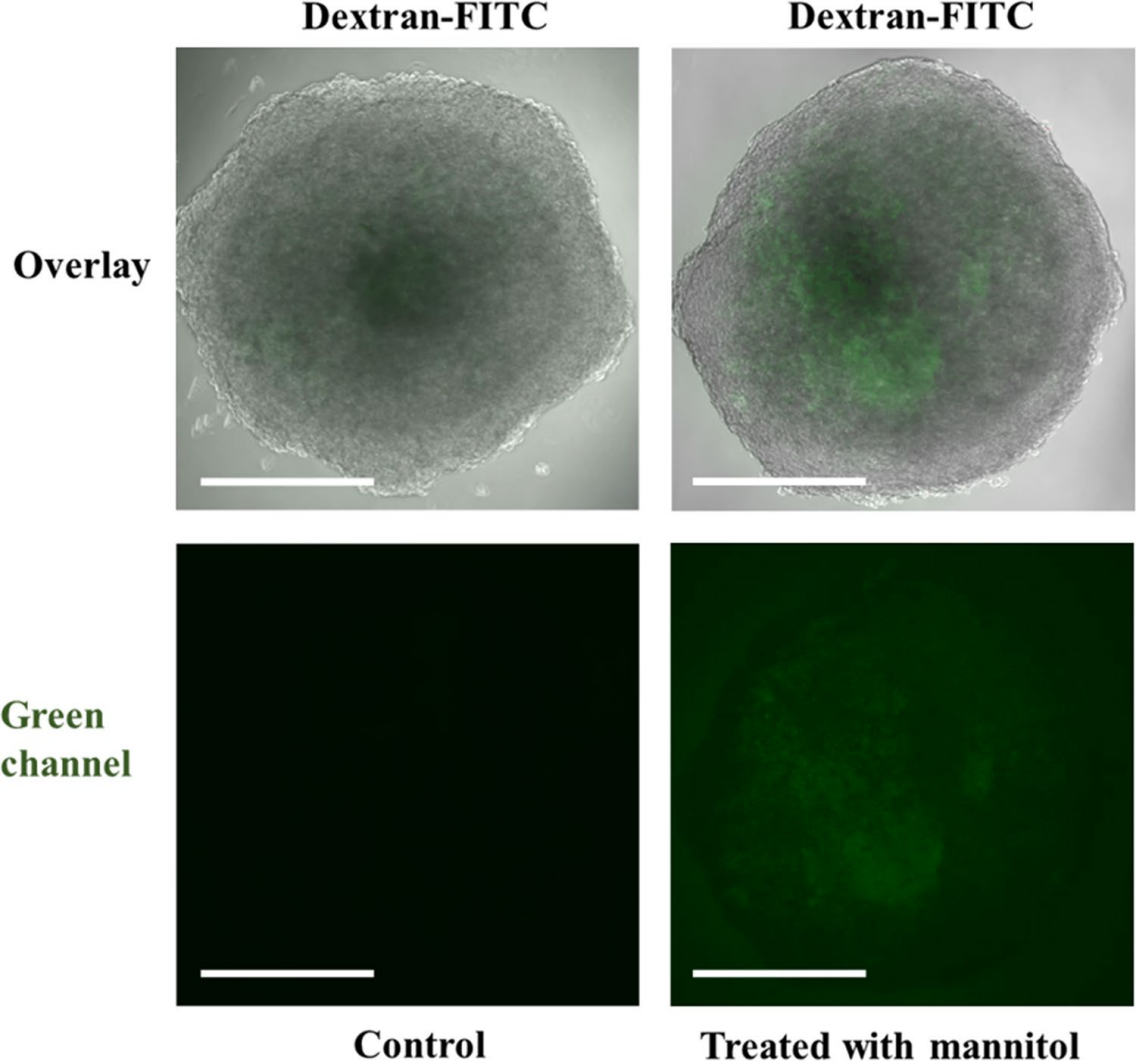
Permeability of brain spheroids with green-fluorescent dextran-FITC (incubation for 30 min). Only after the addition of mannitol (BBB opening agent), dextran-FITC was able to enter the spheroid, demonstrating the intact blood–brain barrier prior to the treatment with mannitol. Scale bars 200 µm.

The results of a permeability assay with the reference compound dextran-FITC (70 kDa, hydrodynamic radius about 12 nm) are shown in Fig. 3. Only after stimulation with mannitol^34^, the BBB became permeable and permitted the entry of dextran^6^. Fluorescently labelled dextran is an accepted model compound to demonstrate the integrity of the BBB^10^.

### Uptake of ultrasmall FAM-labelled gold nanoparticles within BBB spheroids

**Figure 4:**
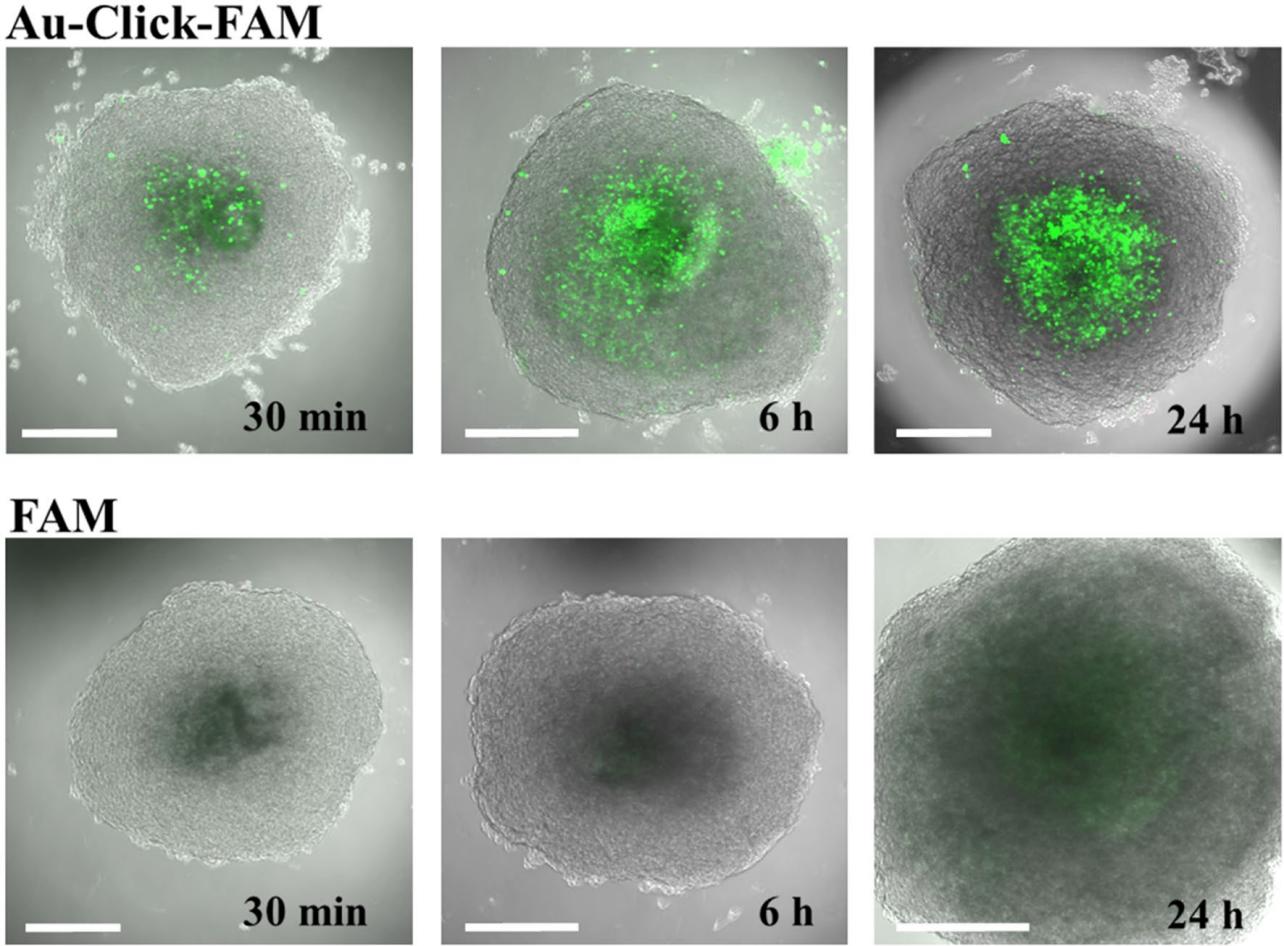
Uptake of ultrasmall FAM-labelled gold nanoparticles (Au-Click-FAM) and dissolved FAM-alkyne by brain spheroids after 30 min, 6 h, and 24 h. The nanoparticles and the dye showed a green fluorescence. The nanoparticles clearly entered the center of the spheroid whereas the dye FAM-alkyne was mostly rejected. Notably, the nanoparticles were mainly present in the center of the organoid and not in the outer pericyte/endothelial cell layer. Scale bars 200 µm.

The permeability of the blood–brain barrier in the spheroids was tested with green fluorescent ultrasmall gold nanoparticles (Au-Click-FAM) and with the dissolved dye (FAM-alkyne) at the same concentration as control, also as a function of time. The nanoparticles were rapidly taken up while the free dye was strongly rejected (Fig. 4). Note that the spheroids were thoroughly washed before imaging in all cases, which removes free and weakly adhering nanoparticles and dissolved FAM-alkyne molecules. Each spheroid was incubated with about 1.8 × 10^13^ nanoparticles (Table 1), i.e. the nanoparticle dose was very high.

**Table 1:** Parameters of gold nanoparticles applied to 3D cell culture models.

*c*(Au stock dispersion)/g/L	0.30
*V*(medium) per well/µL	100
*V*(Au stock dispersion) per well/µL	5
*m*(Au) per well/µg	1.50
*c*(Au) in a well/g/L	0.014
Particle molar concentration (*d* = 2 nm) in each well/µmol/L	0.29
Particle number concentration (*d* = 2 nm) in each well/L	1.73 × 10^17^
Particle number (*d* = 2 nm) in each well	1.82 × 10^13^
Dye molecules in each well	1.64 × ·10^14^

For dissolved FAM-alkyne as control, the same molecular concentrations were used. The gold nanoparticles had a diameter of 1.57 ± 0.62 nm by DCS (lower than the core diameter due to the special characteristics of DCS with ultrasmall particles; see Ref.^54^), of 2.28 ± 0.32 nm by TEM, and carried 9 FAM molecules per nanoparticle. Dynamic light scattering (DLS) was not possible due to the small particle size. Based on ^1^H-DOSY results of similar nanoparticles, we estimate the hydrodynamic diameter to 3–4 nm (see Ref.^30^ for a full characterization of the particles).

**Figure 5:**
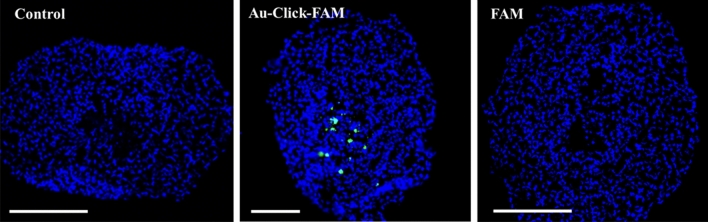
CLSM images of 2D cryosections of brain spheroids after 24 h of incubation with green-fluorescent FAM-labelled gold nanoparticles (Au-Click-FAM) and dissolved FAM-alkyne. The spheroids were cryopreserved in liquid nitrogen, sectioned (thickness 7 µm) and stained with DAPI for 10 min. Scale bars 200 µm.

The images in Fig. 5 show the accumulation of fluorescence after washing and fixation of the spheroids, i.e. the nanoparticles that had been present outside the spheroids during the incubation were removed. In support of the CLSM images, this is a direct proof that the nanoparticles (hydrodynamic diameter 3–4 nm) entered the interior of the spheroid and that the dissolved dye (hydrodynamic diameter about 1 nm) was mostly rejected.

**Figure 6:**
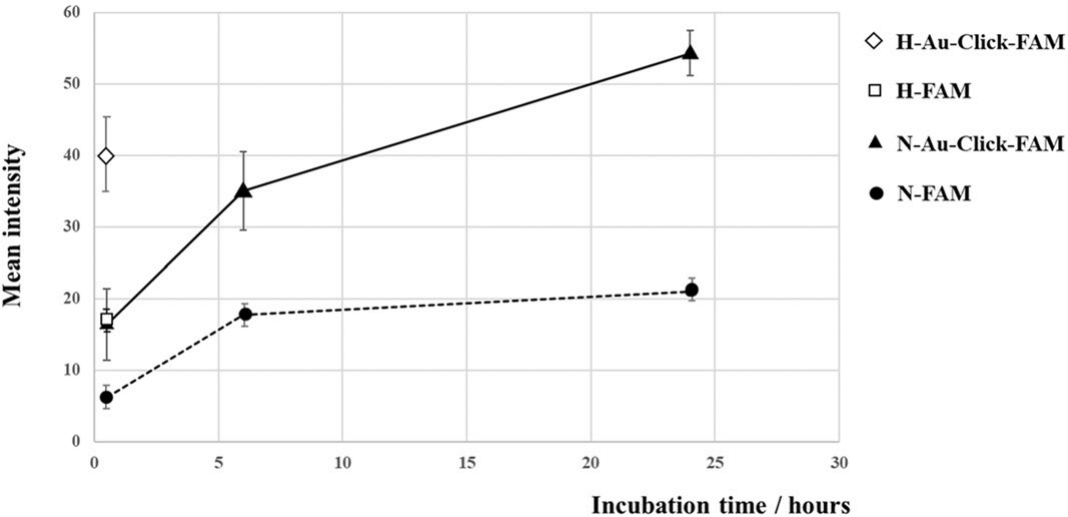
Determination of the green fluorescence intensity inside the brain spheroids during the uptake of ultrasmall gold nanoparticles and FAM-alkyne molecules after 30 min, 6 h and 24 h of incubation under normal oxygen pressure (N-Au-Click-FAM and N-FAM) and after hypoxia, followed by incubation with either ultrasmall gold nanoparticles or FAM-alkyne molecules for 30 min (H-Au-Click-FAM and H-FAM). The fluorescence intensity was integrated and averaged from five spheroids.

As evident from Fig. 4, the nanoparticle concentration inside the spheroid increased over time. This was quantified by integration of the green fluorescence intensity inside the spheroid (Fig. 6). We observed that the uptake of nanoparticles with increasing incubation time was clearly enhanced, e.g. the mean fluorescence intensity increased from 16 (after 30 min) to 54 (after 24 h of incubation). It was also found that FAM-alkyne molecules alone were taken up only to small extent with a mean fluorescence intensity between 6 and 20, depending on the incubation time.

In general, the particle uptake will be driven by the concentration gradient between the outside nanoparticle dispersion (about 1.7 × 10^17^ nanoparticles per L; Table 1) and the interior of the spheroid. However, diffusion along the concentration gradient is prevented by the intact blood–brain barrier function of the spheroids, otherwise the dissolved dye would also diffuse inside (and definitely much faster than the nanoparticles).

**Figure 7:**
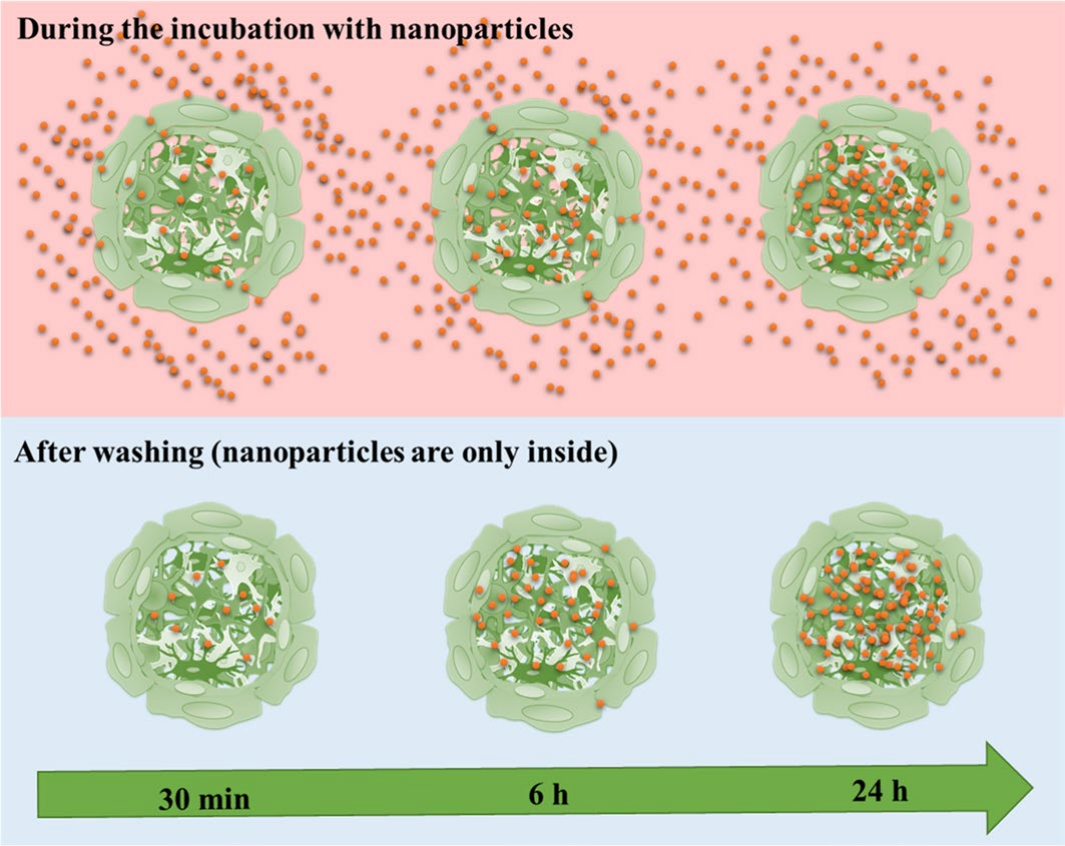
Model for the uptake of nanoparticles across the blood–brain barrier in brain spheroids. The blood–brain barrier slows the entry of nanoparticles. The uptake of nanoparticles is driven by the concentration gradient, i.e. the concentration of nanoparticles outside the spheroid (in dispersion) is always larger than inside the spheroid. As soon as a nanoparticle has entered, it can rapidly diffuse towards the center of the spheroid. Note that all images shown above were recorded with brain spheroids after washing, i.e. they represent the situation in the bottom panel.

The fact that the nanoparticles were only found in the center the spheroid but not in the outer pericyte/endothelial cell layer (Fig. 4) is surprising. Obviously, diffusion alone would lead to a concentration gradient within the spheroid and could not explain the almost empty outer layer. A possible explanation is the presence of a functional blood–brain barrier in the outer pericyte/endothelial cell layer that slows the entry of molecules and nanoparticles. Here we propose the following tentative model: After the blood–brain barrier has been overcome, the nanoparticle is rapidly transported across the pericyte/endothelial cell layer into the center of the spheroid. Inside, the diffusion is faster and encounters less resistance in part due to greater spacing between the cells of the spheroid core vs the tight junctions of the BBB surface. If the internal diffusion is faster than the “waiting time” at the outer surface of the spheroid (outside the blood–brain barrier), it can explain the distribution of the nanoparticles. It is also clear from Fig. 4 that the weakly adhering nanoparticles are removed from the pericyte/endothelial cell layer during the washing process. The fact that we see nanoparticles in the center of the spheroid after 5 min suggests that the transport occurs by rapid diffusion after the nanoparticles have started to enter the spheroid. This time-span is probably too short to permit endocytosis, exocytosis, and transcytosis. Figure 7 shows our tentative model to visualize this mechanism.

**Figure 8:**
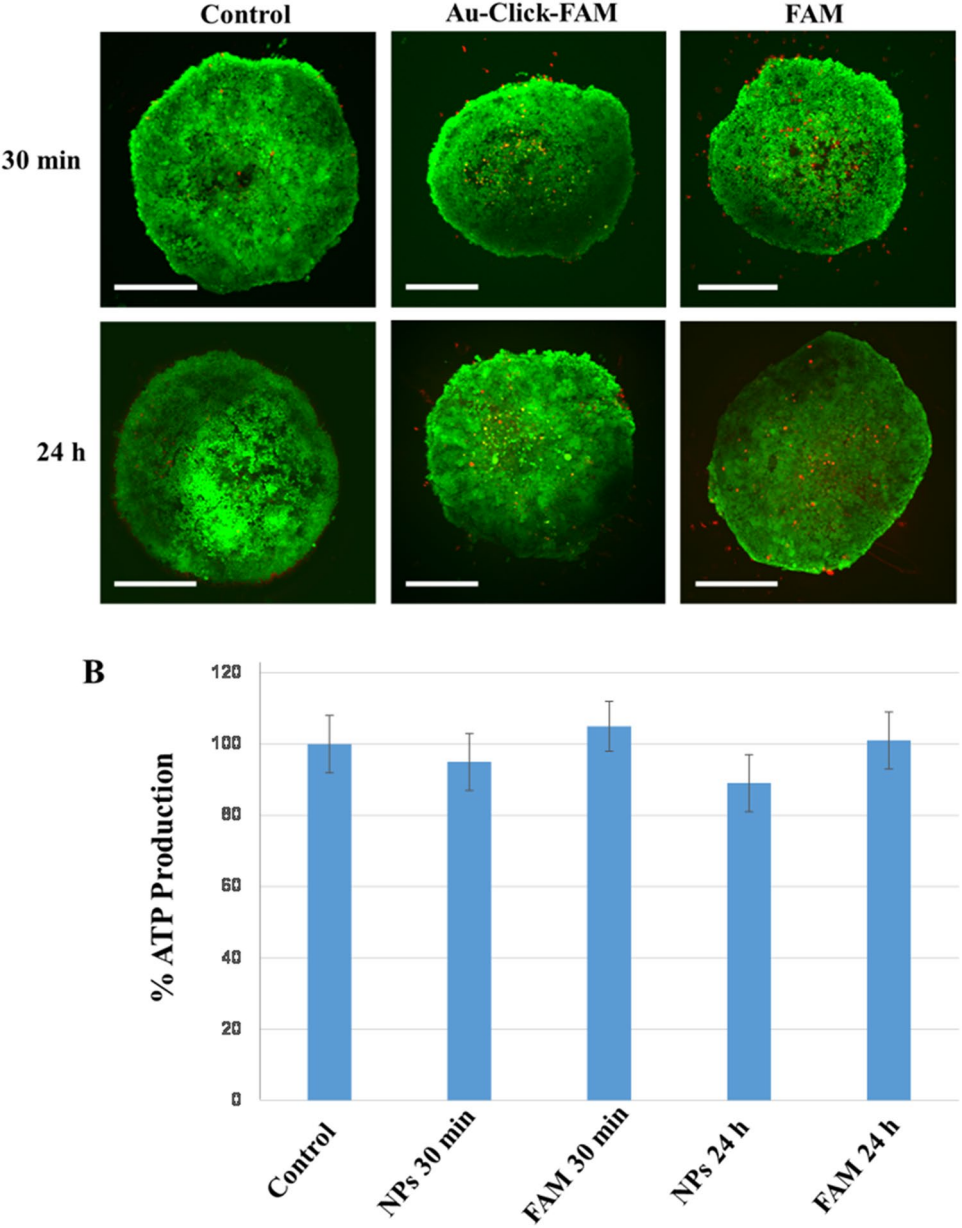
(**A**) Live/dead assay of brain spheroids, incubated with Au-Click-FAM ultrasmall gold nanoparticles or FAM-alkyne molecules. Scale bars 200 µm. The green fluorescence shows living cells, the red fluorescence shows dead cells. (**B**) ATP production in brain spheroids after treatment with Au-Click-FAM nanoparticles (NPs) or dissolved FAM-alkyne (100%: ATP production in untreated spheroids). There was no significant difference in the ATP production after 30 min and 24 h.

The nanoparticle toxicity was assessed by measuring the cell viability via the ATP production. Neither treatment with ultrasmall FAM-labelled gold nanoparticles nor treatment with dissolved FAM-alkyne alone for 30 min or 24 h significantly reduced the ATP production compared to the control. Thus, we can assume that the viability of the spheroids was not compromised by the nanoparticles or the dye, i.e. neither the nanoparticles nor the dissolved dye were harmful to the spheroids (Fig. 8).

### Uptake of nanoparticles after hypoxia treatment of BBB speroids

**Figure 9:**
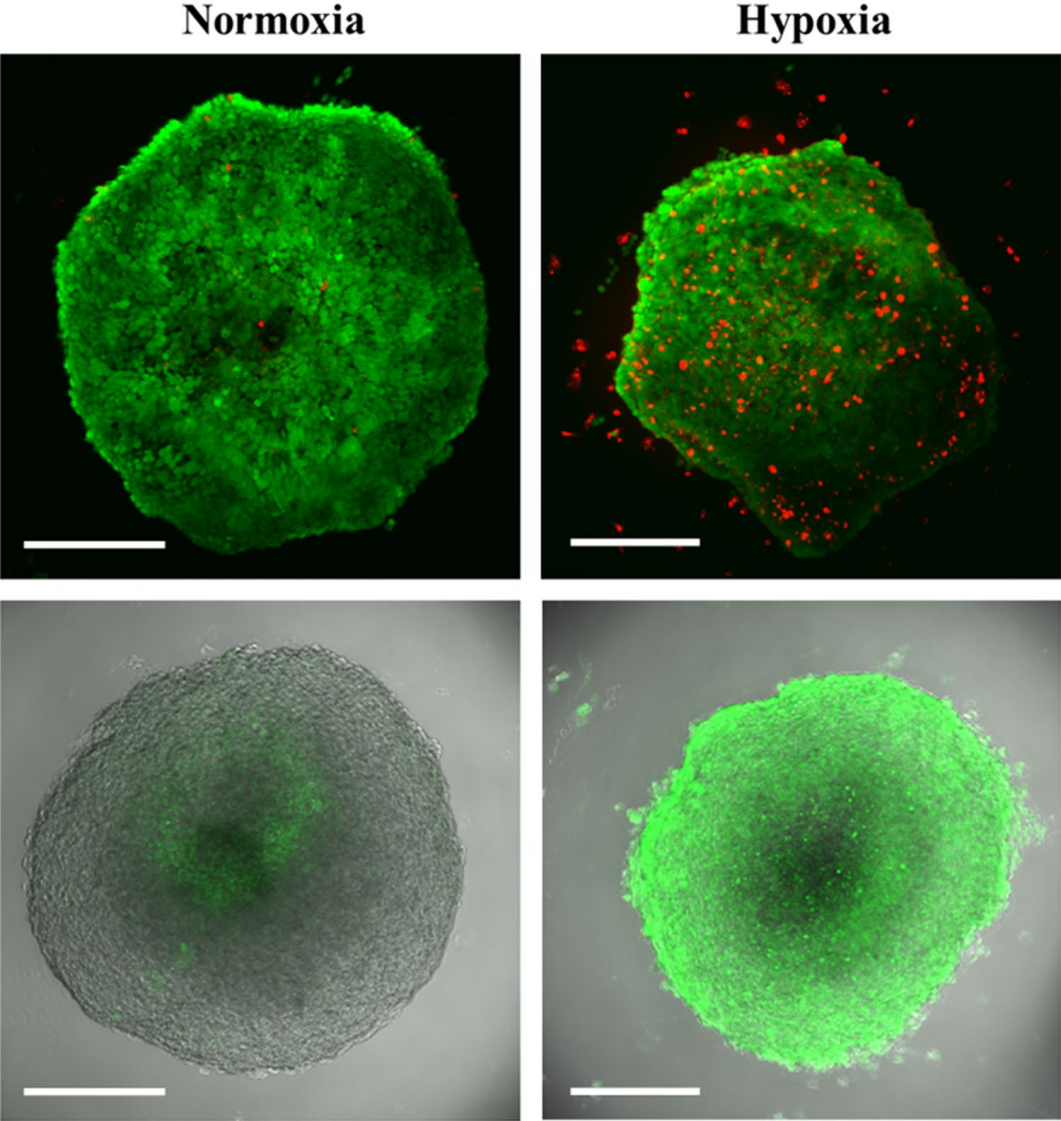
Brain spheroids after 24 h hypoxia. Top: Live-dead assay with calcein AM (green; living cells) and ethidium (red; dead cells). Bottom: Fluorescence signal from Image-iT Green Hypoxia Reagent. Scale bars 200 µm.

It is known that the blood–brain barrier can be damaged by hypoxia, e.g. after an ischemic stroke^35–37^. To simulate this process, we have subjected brain spheroids to 24 h of hypoxia, followed by 30 min incubation with nanoparticles. Figure 9 shows the effect of hypoxia on the spheroids. An increased number of dead cells is evident, although most of the cells remain viable and impermeable to ethidium in the live/dead assay stain. A hypoxia staining is strongly visible after incubation of spheroids in the hypoxia chamber.

**Figure 10:**
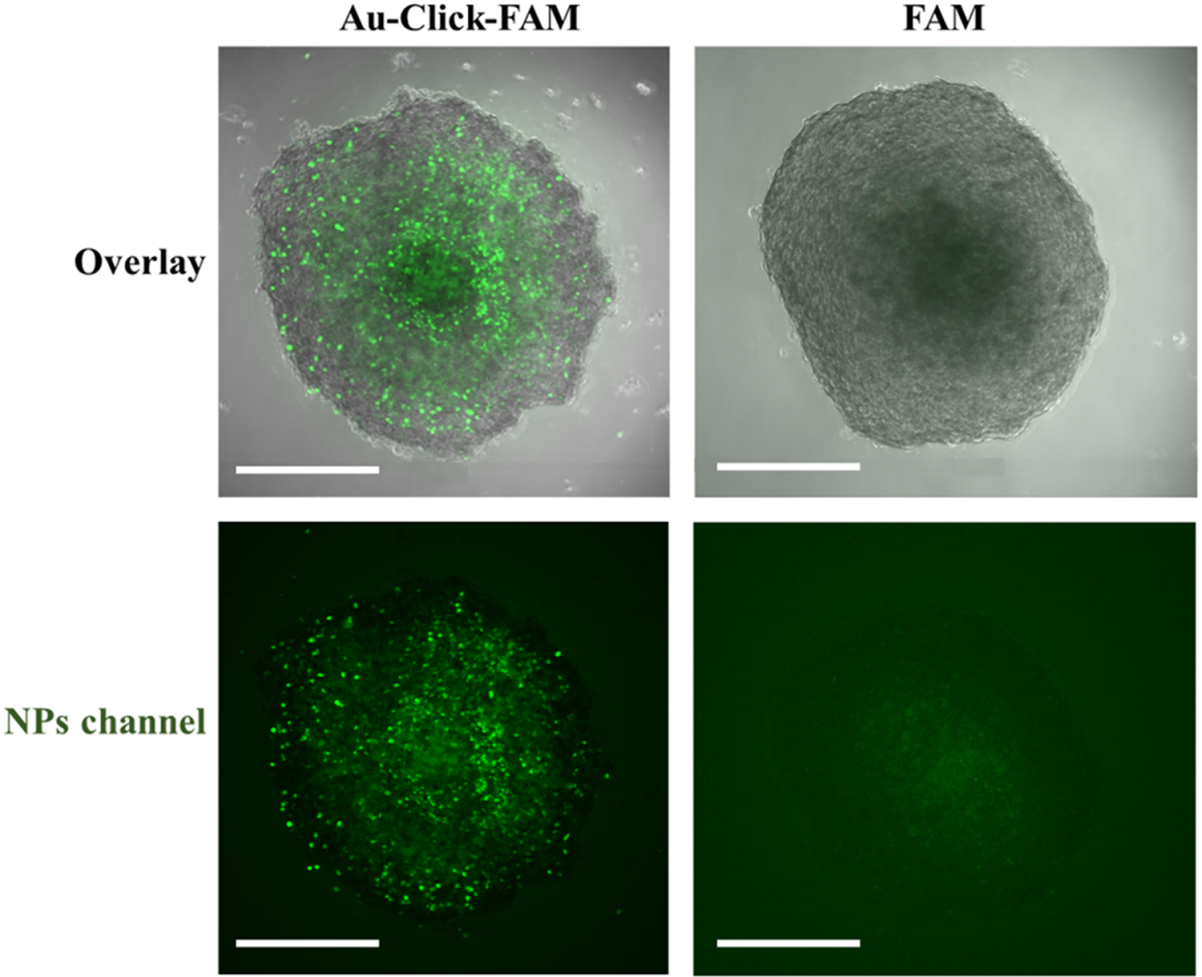
Effect of hypoxia on the uptake of Au-Click-FAM nanoparticles or FAM-alkyne after 30 min incubation after 24 h hypoxia. The nanoparticles entered the spheroid and were also found in the external pericyte/endothelial cell layer. The dissolved FAM-alkyne molecules also entered the spheroid to a considerable extent. This demonstrates the hypoxia-induced damage to the blood–brain barrier. Scale bars 200 µm.

After hypoxia, the uptake of nanoparticles was clearly enhanced (Fig. 10). The mean fluorescence intensity was 40 on the same relative scale shown for nanoparticle uptake at 30 min in Fig. 6 (in contrast to 16 under normoxia). It is also evident that the particles were now also present in the pericyte-endothelial cell layer because the blood–brain barrier had been compromised. In contrast to normoxic spheroids, the dissolved FAM-alkyne dye now also entered the spheroid (relative fluorescence intensity 17 on the same scale as in Fig. 6). This shows that the in vitro blood–brain barrier in the brain spheroids is more permeable after hypoxia, in line with the clinical experience that a stroke severely compromises the blood–brain barrier function^35,38,39^.

## Discussion and conclusions

One of the most challenging goals in pharmacological research is the passage through the blood–brain barrier to deliver drugs to the central nerve system (CNS). The use of nanoparticles as a delivery system for biomolecules may enhance the drug transport through the BBB in neurodegenerative diseases. Different inorganic nanoparticular carriers have been applied in this field, e.g. iron oxide, cerium oxide, molybdenum, silica or gold nanoparticles^40–44^. Compared to other types of nanoparticles, gold nanoparticles have received considerable interest because of their relative low cytotoxicity, predominant optical properties for imaging, well-established syntheses, and their potential to cross the BBB^14,28,45,46^.

The penetration mechanism across the BBB depends on the nanoparticle size and composition. Transmembrane diffusion is mostly observed for low molecular weight and high lipid solubility molecules^47^. Some biomolecules, such as transferrin, are able to cross the BBB via a receptor-mediated process^48^. Sela et al. have shown a spontaneous migration of gold nanoparticles with the size 2.5 nm through the blood brain barrier^49^. Betzer et al*.* synthesized insulin-coated gold nanoparticles with different sizes (20, 50 and 70 nm) and studied the biodistribution and their ability to cross the BBB in Balb/C mice^50^. It was shown that 2 h after injection, 20 nm gold nanoparticles had a broad biodistribution and a considerable accumulation within the brain. The possibility to apply steady and alternating magnetic fields to drive magnetic nanoparticles and liposomes across the BBB was also shown^21,40,51^.

Here, ultrasmall gold nanoparticles were readily taken up by six-cell human brain spheroids. The blood–brain barrier, formed by an external layer of pericytes and endothelial cells, was intact as demonstrated by the rejection of dextran-FITC, consistent with earlier reports^10^. The nanoparticles (about 2 nm in core diameter, hydrodynamic diameter 3–4 nm) were able to enter the brain spheroid, driven by the outside-inside concentration gradient. However, the blood–brain barrier regulated the particle uptake. This regulation (i.e. the opening of the blood–brain barrier) was slower than the subsequent diffusion into the center of the spheroid. This led to an accumulation of the particles in the center of the spheroid while only few particles were observed during the passage across the pericyte/endothelial cell layer. In contrast, the dissolved FAM dye was able to enter the spheroid only to a small extent, indicating that ultrasmall nanoparticles are preferentially crossing the blood–brain barrier by a hitherto unknown transport mechanism. A prolonged hypoxia for 24 h compromised the blood–brain barrier so that both the nanoparticles and the dissolved dye could enter more easily.

We have used 70 kDa FITC-dextran as reference that has often been used as model compound to check the integrity of the BBB^6,10^. Our results show that the BBB is intact in the spheroid. 70 kDa dextran has a hydrodynamic diameter of about 12 nm. Thus, it is larger than the gold nanoparticles (metal core diameter 2 nm; hydrodynamic diameter 3–4 nm)^30^, therefore dextran and gold nanoparticles do not have the same size. However, the observation that the nanoparticles are entering the spheroids is independent from the size of the dextran used. Unfortunately, no information can be derived from the light microscopic images about a possible agglomeration of the nanoparticles. Their diameter of about 2–4 nm (core diameter/hydrodynamic diameter) is much less than the resolution of the confocal laser scanning microscope. Therefore, we cannot distinguish individual particles. However, the particles have a size which is comparable to the dissolved FAM dye (a few nm). The organoids were washed in the same way in all cases, i.e. if the dye had been washed out, the nanoparticles would have vanished as well.

In summary, ultrasmall gold nanoparticles are able to cross the blood–brain barrier in these in vitro human brain cell spheroids, consisting of six different cell types. This is of high interest, e.g. for future therapies to deliver functional drugs into the brain. Together with our earlier observation that such nanoparticles can enter cells and also the cell nucleus (in contrast to the dissolved dye)^28,30^, this opens a potential pathway for targeted therapies inside the brain which are not possible with water-dissolved drugs. However, while the toxicity was very low in this model, a study of cumulative injury in animal models will be required to better predict applications in humans, since the clearance kinetics of the particles are still unknown.

## Methods

### Chemicals

Elemental gold (≥ 99%) was dissolved in *aqua regia* to prepare tetrachloroauric acid (HAuCl_4_). We used sodium borohydride (NaBH_4_, ≥ 96%; Sigma-Aldrich), sodium l-ascorbate (≥ 99%; Sigma-Aldrich), copper(II)sulfate pentahydrate (≥ 99%; Sigma-Aldrich), tris(3-hydroxypropyl-triazolyl-methyl)amine (THPTA, ≥ 95%; Sigma-Aldrich), dipotassium hydrogen phosphate and potassium phosphate (p.a.; Panreac Applichem), amino guanidine hydrogen carbonate (≥ 98%; Alfa Aesar) fluorescein alkyne (≥ 95%, FAM-alkyne; 5-isomer; Lumiprobe), and the tripeptide 6-azido-lysine-cysteine-asparagine K(N_3_)CD (≥ 95%; EMC Microcollections, Tübingen, Germany). FITC-labelled dextran (fluorescein isothiocyanate-dextran) was obtained from Sigma-Aldrich with a molecular weight of 70 kDa (hydrodynamic diameter 12 nm). Ultrapure water with a specific resistivity of 18.2 MΩ was prepared with a Purelab ultra instrument (ELGA) and used for all synthesis and purifications unless otherwise noted. Before all syntheses involving nanoparticles, we cleaned all glassware with boiling *aqua regia* and washed it thoroughly washed afterwards.

### Instruments

UV–Vis spectroscopy was performed in Suprasil quartz glass cuvettes with a sample volume of 600 μL with a Varian Cary 300 spectrometer. Differential centrifugal sedimentation was done with a CPS Instruments DC 24,000 disc centrifuge (24,000 rpm). A density gradient was formed with two sucrose solutions (8 wt% and 24 wt%) and capped with 0.5 mL dodecane as stabilizing agent. A poly(vinyl chloride) (PVC) latex in water with a particle size of 483 nm provided by CPS Instruments served as calibration standard. The DCS calibration was carried out prior to each run. A sample volume of 100 μL of dispersed nanoparticles was used. The recording time was about 6 h at the given centrifugation speed. For elemental analysis (gold and copper), the nanoparticles were dissolved in *aqua regia* and analyzed by atomic absorption spectroscopy (AAS; Thermo Electron M-Series spectrometer; graphite tube furnace according to DIN EN ISO/IEC 17025:2005). Confocal laser scanning microscopy (CLSM) was carried out with an Olympus Fluoview Fv10i (Olympus, Tokyo, Japan) microscope.

### Synthesis of clicked nanoparticles

The synthesis and click reaction of the ultra-small gold nanoparticles were performed as reported earlier^28,30^. Briefly, the ultrasmall gold nanoparticles were produced by a modified one-phase Brust synthesis, and subsequently a copper-catalyzed azide-alkyne cycloaddition (CuAAC) was performed^52,53^. For gold nanoparticles terminated with azide groups, the cysteine- and azide-containing peptide K(N_3_)CD (4.5 µmol) was dissolved in 6 mL water and the pH was adjusted to 7 by adding 0.1 M sodium hydroxide solution while stirring continuously. The solution was degassed with argon and 30 µL 50 mM tetrachloroauric acid (HAuCl_4_, 1.5 µmol) was added. After the yellow color of the HAuCl_4_ had disappeared, 22.5 μL of a 200 mM ice-cold sodium borohydride solution (NaBH_4_, 4.5 µmol) was injected into the flask. After adding NaBH_4_, the solution quickly turned brown and the dispersion was stirred at room temperature for another hour.

For CuAAC, the dispersion of unpurified azide-terminated gold nanoparticle was treated as follows: 112.5 µL of 20 mM alkyne fluorophore (FAM-alkyne) were added to the azide-terminated nanoparticles. 2.7 mL of a 20 mM solution of aminoguanidine in potassium phosphate buffer (0.05 M, pH 8) were added to the reaction mixture to avoid the side reactions of reactive carbonyl compounds. 600 μL of this resulting solution was injected into a 1:5 (mol:mol) mixture of 20 mM copper(II) sulphate and 50 mM THPTA. The final addition of 500 μL of 100 mM sodium ascorbate solution led to the in-situ formation of the catalyzing Cu(I) complex for the click reaction. The solution was stirred overnight under argon at room temperature. The nanoparticles were purified by passing the dispersion through an ultrafiltration spin column (MWCO 3 kDa, 15 mL; Amicon; Merck) for 20 min at 14,000×*g* to remove all unreacted compounds with the filtrate. After a number of centrifugation steps, the filter with the nanoparticles was rinsed with potassium phosphate buffer (0.05 M, pH 8) until the filtrate was clear, i.e. all molecular impurities had been removed. The gold nanoparticles were recovered from the filter by sampling with a pipette (volume about 1 mL).

### Analysis of the dispersed nanoparticles

The concentration of gold nanoparticles was determined by atomic absorption spectroscopy. The molar gold concentration gave the number concentration of nanoparticles, using the density of elemental gold (19,300 kg/m^3^), the assumption of spherical nanoparticles and an average diameter of 2 nm as determined earlier by HRTEM^30^. The concentration of the clicked FAM-alkyne was determined by quantitative UV spectroscopy and fluorescence spectroscopy after a calibration with the free fluorophore-labelled ligands at the following wavelengths: FAM-alkyne (*λ*_Ex_ = 485 nm). Based on a particle diameter of 2 nm (solid core by HRTEM), 9 FAM-alkyne molecules per nanoparticles were determined by UV–Vis spectroscopy, i.e. slightly more than in an earlier synthesis^30^. For control experiments, the same concentrations of dissolved FAM-alkyne molecules as delivered in gold-conjugated form were applied. All nanoparticle parameters and concentrations are summarized in Table 1.

### Cells and spheroids

The spheroids were prepared according to Nzou et al*.*^8,33^. Spheroids containing six brain cell types, i.e. 30% primary human brain microvascular endothelial cells (HBMEC; from Cell Systems, Kirkland, WA), 15% primary human brain vascular pericytes (HBVP; from ScienCell Research Laboratories, Carlsbad, CA), 15% primary human astrocytes (HA; from ScienCell Research Laboratories), 5% human iPSC-derived microglia cells (HM from Tempo Bioscience Inc., San Francisco, CA), 15% human iPSC-derived oligodendrocyte progenitor cells (HO; from Tempo Bioscience Inc., San Francisco, CA), and 20% human iPSC-derived cortical neurons (HN; from ATCC, American Type Culture Collection, Manassas, VA) were used to make a total of approximately 3000 cells per spheroid.

Spheroids containing HA, HM, HO and HN formed in a 50:50 mixture of astrocyte medium without astrocyte growth supplements and neural maintenance-XF medium under normal growth conditions for 48 h in ultra-low attachment plates (Corning). The medium was mixed with heat-inactivated fetal bovine serum (FBS; Thermo Fisher) and 10 ng/μL rat tail collagen I (Corning). Subsequently, we added HBMEC and HBVP to coat the neural-glial spheroid. The spheroids were cultured under normal growth conditions in 60% neural maintenance-XF medium, 20% astrocyte medium, and 20% endothelial cell growth medium (EGM, Lonza). The spheroids were matured for 48 h and put into 96-well plates for long term cultivation. The spheroids that were used in the experiments had been cultivated for 7 to 8 days.

### Cell viability

The cell viability was evaluated with a Molecular Probes Live-Dead cell reagent system (Thermo Fisher). Briefly, human brain spheroids were collected for cell viability analysis and incubated at room temperature for 30 min in spheroid medium containing 2 μM calcein AM (Invitrogen, green: live cells) and 4 μM ethidium homodimer-1 (Invitrogen, red: dead cells). After washing 3 times with phosphate buffered saline (PBS), the spheroids were imaged by CLSM.

### Immunohistochemistry

Spheroids were transferred to 1.7 mL Eppendorf tubes, fixed in 4% formaldehyde (Polysciences Inc, Warrington, PA) for 30 min at 4 °C, and washed three times with cold PBS. The spheroids were then permeabilized with 0.1% Tween-20 in phosphate buffered saline (PBS) for 10 min at TR and washed three times with cold PBS. Then, they were treated with Protein Block (Dako Group, Troy, MI) for 2 h at room temperature. Then, they were incubated at 4 °C overnight in Antibody Diluent (Dako) solution which contained the primary antibodies anti-ZO-1 and anti-CD31 (1:200, Corning). The spheroids were subsequently washed three times with cold PBS and incubated with AF488 goat-anti-mouse IgG (1:500, Life Technologies) overnight in antibody diluent (Dako) at 4 °C. Nuclear staining was performed by incubation of the spheroids with DAPI (1:1000) in PBS for 10 min. The spheroids were washed and imaged using CLSM. For imaging, at least randomly selected spheroids were randomly selected for each stain.

### Permeability assay

One group of spheroids was incubated in media under normal growth conditions with dextran-FITC for 30 min. A control group was treated with 1 M mannitol solution^34^. 10 min prior to incubation with dextran-FITC. The spheroids were washed three times before imaging with the CLSM.

### ATP production

This was done as described earlier^8^. We transferred the spheroids ito an opaque-walled 96-well plates and added 100 µL medium to each well. The CellTiter-Glo Reagent was prepared by mixing CellTiter-Glo Substrate and CellTiter-Glo Buffer (Promega Life Sciences, Madison, Wisconsin) according to the manufacturer's instructions. 100 µL CellTiter-Glo Reagent were added to 100 μL of medium with the spheroids. Cell lysis was induced by strong mixing in an orbital shaker. The plate was incubated at room temperature for 10 min to stabilize the luminescent signal. The luminescence was measured with a microplate luminometer (Veritas, Mountain View, CA), followed by background luminescence subtraction.

### Nanoparticle uptake studies

After 7 to 8 days of multicellular BBB spheroid generation, gold nanoparticles or the corresponding control (dissolved FAM-alkyne) were added and incubated for the indicated time periods, i.e. 30 min, 6 h, 24 h for normoxia and 30 min for hypoxia, at 37 °C (Table 1). Then, the spheroids were washed three times with PBS and immediately analysed by CLSM. For cryosections, the spheroids were cryopreserved in liquid nitrogen and sectioned (thickness of 7 µm) and stained with DAPI (1:1000) for 10 min. The sections were the analysed by CLSM.

### Hypoxia

At day 7, a 96-well plate containing spheroids was incubated with medium (60% neuronal maintenance medium, 20% endothelial growth medium, and 20% astrocyte growth medium) containing the gold nanoparticles and/or the corresponding control (dissolved FAM-alkyne) and/or Invitrogen Image-iT Green Hypoxia Detection Reagent (Thermo-Fisher) at 37 °C, 0.1% O_2_, 99.9% N_2_ for 24 h in an Xvivo System G300C (BioSpherix, Redfield, NY, USA) to investigate the effect of the hypoxia on the gold nanoparticle permeability and distribution. Spheroids grown under normoxic conditions and spheroids grown under hypoxic condition were collected, washed and analysed immediately by CLSM.

### Statistics

Data are expressed as the average ± standard deviation of the mean for each group. Student’s *t*-test was used to compare groups; *p* values less than 0.05 were considered as significant.

## Data Availability

The datasets generated during and/or analysed during the current study are available from the corresponding author on reasonable request.
